# Perception of quality of care among residents of public nursing-homes in Spain: a grounded theory study

**DOI:** 10.1186/1471-2318-13-65

**Published:** 2013-06-28

**Authors:** Beatriz Rodríguez-Martín, María Martínez-Andrés, Beatriz Cervera-Monteagudo, Blanca Notario-Pacheco, Vicente Martínez-Vizcaíno

**Affiliations:** 1 Health and Social Research Center, University of Castilla-La Mancha, Cuenca, Spain; 2 Faculty of Occupational Therapy, Logopedia and Nursing, University of Castilla-La Mancha, Talavera de la Reina, Spain

**Keywords:** Nursing Home, Qualitative Research, Quality of Health Care, Patient Satisfaction, Aged

## Abstract

**Background:**

The quality of care in nursing homes is weakly defined, and has traditionally focused on quantify nursing homes outputs and on comparison of nursing homes’ resources. Rarely the point of view of clients has been taken into account. The aim of this study was to ascertain what means “quality of care” for residents of nursing homes.

**Methods:**

Grounded theory was used to design and analyze a qualitative study based on in-depth interviews with a theoretical sampling including 20 persons aged over 65 years with no cognitive impairment and eight proxy informants of residents with cognitive impairment, institutionalized at a public nursing home in Spain.

**Results:**

Our analysis revealed that participants perceived the quality of care in two ways, as aspects related to the persons providing care and as institutional aspects of the care’s process. All participants agreed that aspects related to the persons providing care was a pillar of quality, something that, in turn, embodied a series of emotional and technical professional competences. Regarding the institutional aspects of the care’s process, participants laid emphasis on round-the-clock access to health care services and on professional’s job stability.

**Conclusions:**

This paper includes perspectives of the nursing homes residents, which are largely absent. Incorporating residents’ standpoints as a complement to traditional institutional criteria would furnish health providers and funding agencies with key information when it came to designing action plans and interventions aimed at achieving excellence in health care.

## Background

Nursing homes are an alternative to private homes for persons who need long-term care but do not wish or are not able to live at home. Since the 60’s, there is growing interest in improving the quality of care in nursing homes, and to attain this goal it is known that measures of quality must be targeted at achieving safe, effective, efficient, person-centered care [1-3]. Analysis of nursing-home quality is focused on quantify nursing home quality results and comparison of nursing homes’ resources [1,4,5].

In the last decade, the understanding of what quality means from the nursing homes’ client perspective has become a growing focus of interest [3,6,7], such that state and federal programs in the United States of America (USA) have included these perspectives in their survey and certification processes [1,3]. Indeed both the Excellence Model of the European Foundation for Quality Management [8] and the USA National Quality Forum [9] advocate the participation of care receivers as a key element for improving quality [2].

Quality of care in nursing homes has been traditionally assessed through the Donabedian approach, using the indicators of structure, process and outcome in the majority of studies [1,10]. Most of the studies aimed to analyze quality of nursing-home care are usually focused on how this construct is perceived by professionals [6,11] or family members [12], especially in the case of institutionalized people with cognitive impairment [5].

Traditionally, the point of view of residents has been measured by using questionnaires that monitor the satisfaction with the care received [7,13]; nevertheless, perception of quality is a more global construct of which satisfaction might be a dimension [14]. Quality of care is a social construct that assumes different forms depending on age, gender, socioeconomic characteristics and health level [1,15]. The exclusive use of surveys to study overall health care quality has some weakness including the tendency to framing the protagonists’ subjective experiences into rigid categories imposed by the researchers on the basis of preconceived ideas. On the other hand, quantitative and qualitative approaches are not necessarily mutually exclusive, one can inform the other [15].

To our knowledge, studies that incorporate the voice of residents to clarify what is quality of care in nursing homes are scarce [13,16-18], and no study was undertaken in a Mediterranean setting, where it’s known that the formal care systems are not well seen, prevailing a hierarchical compensation family model when people choose the type of care system they prefer [19,20]. Moreover, no study has included relatives of cognitively impaired persons in the sample, despite the recommendations for such persons to be included in quality assessments [5].

Accordingly, this study sought to ascertain what quality of care meant to residents in nursing homes.

## Methods

### Design

We used in-depth interviews and grounded theory dimensional analysis to collect and analyze the data [21-23]. This inductive method was chosen so as to obtain a theoretical explanation by analyzing participants’ conceptualizations of quality of care. In the grounded theory paradigm, the theory is conceived as a collection of well-developed concepts linked by linking sentences which together constitute an integrated framework that can be used to explain or predict phenomena [22].

### Participants

Data of this study come from a broader research project (Qualitative analysis of formal care in public nursing homes) that analyzes the phenomenon of institutionalization of older persons. The participants and methods have been described extensively elsewhere [24]. In brief, in-depth interviews were conducted using a theoretical sample of 20 persons living at a public nursing home in Talavera de la Reina (Spain), aged 65 years and over, with no cognitive impairment; eight relatives of residents with cognitive impairment were also interviewed. Spain has 5393 nursing homes, classified by type of funding in public, private, or concerted. Currently, 26.6% of them are public funding facilities. These nursing homes provides supervision or assistance with activities of daily living, services of junior nursing staff, nurses, doctor, occupational therapist, speech therapist, social worker and recreational assistance. The study sample comes from a nursing home where 180 assisted and unassisted older people live. The residents pay 75% of their net income for their accommodation, and the allocation of staff and equipment is regulated by current Spanish legislation. In Spain, these types of institutions are generally standardized in terms of amenities and staff [25]. We used theoretical sampling during the process of data analysis, so that each new case was selected because he/she was particularly appropriate for clarifying relationships and logic among constructs. Sampling continued until the saturation of information had been reached, the point at which enlarging a sample ceases to furnish new analytical concepts [23,26]. Informants of both sexes, different age groups and sociodemographic characteristics were included (Tables 1 and 2), in an effort to maximize opportunities to discover dissimilarities among concepts, and to make denser categories in terms of its properties and dimensions [22,27].

**Table 1:** Sociodemographic characteristics of the sample: residents without cognitive impairment (n = 20)

**Variables**		**Women**	**Men**
Age	65-75 years	2	3
	76-85 years	3	3
	86-95 years	6	3
Marital status	Married	1	1
	Single	1	3
Educational level	Widowed	9	4
	Separated	0	1
	Unable to read and write	3	0
	Able to read and write	6	6
Previous institutionalization	Primary education completed	2	3
	Yes	2	2
	No	9	7
Date of admission	1 to 5 years ago	5	5
	6 to 10 years ago	4	3
	Over 10 years ago	2	1

**Table 2:** Sociodemographic characteristics of the sample: residents with cognitive impairment (n=8)

**Variables**		**Women**	**Men**
Resident’s age	65-75 years	1	1
	76-85 years	1	1
	86-95 years	2	2
Age of next-of-kin interviewee	45 to 55 years	2	1
	56 to 65 years	2	1
	Over 65 years	0	2
Next-of-kin interviewee relationship with the resident	Daughter/Son	3	2
	Spouse	1	2
Resident’s marital status	Married	0	2
	Widowed	4	2
Resident’s educational level	Unable to read and write	0	0
	Able to read and write	4	4
	Primary education completed	0	0
Previous institutionalization	Yes	2	4
	No	2	0
Date of admission	1 to 5 years ago	1	3
	6 to 10 years ago	2	1
	Over 10 years ago	1	0

Inclusion criteria for informants were persons aged 65 years and over, living at the nursing home for at least three months, the minimal estimated time to get an accurate picture of facilities, services and staff. Residents admitted on a temporary-stay basis were not considered eligible because short-term residents may have different needs and characteristics [1]. In the case of residents whose degree of cognitive impairment prevented them from directly participating in an interview (score higher than two on Pfeiffer’s Short Portable Mental Status Questionnaire) [28], the closest family member to the resident (proxy) was interviewed instead of him. Since such relatives were well acquainted with the personal history of the resident’s life, his/her habits and preferences, they were deemed more appropriate than professional caregivers in terms of understanding the resident’s views on quality.

### Data collection

Interviews were held by appointment and were conducted into the nursing home in a peaceful and quiet place. Each interview started out with an open-ended question about the personal experience of care at nursing home, which elicited subjective responses about their perceptions. The interviewer, the main researcher, had a topic list that could emerge openly throughout the interviews, but not necessarily at each interview (Table 3). This topic list was refined and concretized guided by theoretical sampling [23]. Interviews were conducted in 2010 and lasted 50 to 120 minutes. All interviews were recorded using a digital recorder, rendered anonymous, and literally transcribed.

**Table 3:** Interview topics list

Background and experience at nursing home	Date of admission
	Prior impression of nursing homes
	Experience in and current assessment of nursing home
	Nursing-home staff
Perceptions and preferences	Delivered care
	Nursing home facilities
	Nursing home activities and services
	Relationships with nursing-home staff
	Relationships with other residents
	Relationships with family members and people outside nursing home

### Ethical considerations

The study was approved by the Clinical Research Ethics Committee of Nuestra Señora del Prado Hospital, in Talavera de la Reina, Spain, and by the management of the nursing home where the study was undertaken. After a full explanation adapted to the research project, all participants were asked to give their informed consent to a sound recording of the interview and its subsequent analysis. In the case of residents with cognitive impairment, the consent of the proxy or, where applicable, the legal guardian was obtained.

### Data analysis

After transcribing the in-depth interviews, the texts were collated and sorted. Using grounded theory methods, three qualitative methodology research experts drawn from different disciplines (Anthropology, Sociology and Nursing) analyzed the transcriptions, with the aim of ascertaining participants’ perception of overall health care quality and obtaining a theoretical explanation for this.

Data-collection, analysis and interpretation were simultaneously undertaken in an interactive process, thus the results of the first data analysis informed subsequent data-collections, thereby enabling key topics to be studied in depth [23]. This implied constantly going back and forth among the interview transcriptions, the analytical memoranda (theoretical ideas about the codes and their relationships) and a review of the literature [27,29].

According to grounded theory principles, our analysis identified specific concepts that explained how informants perceived overall quality of nursing home care. These concepts were labeled and classified into categories using open, axial and selective coding processes [29]. Firstly, each of the three persons in the analysis team separately performed an open coding process, labeling the concepts that emerged from the interviews and sorting these into categories. Once this individual coding had been completed, the process was then repeated on a joint basis and the previously identified categories reorganized. The analysis team shared their research notes and hypotheses, which helped them reach a consensus on new categories and hypotheses, and improve their comprehension of the texts. Finally, an axial and selective coding process was performed jointly. Thus, while open coding fractured the data in short units of significance, axial coding connected them into categories and subcategories in a hierarchical order, and finally selective coding integrated the categories to build a substantive theory. This theory described the relationship among a set of categories that emerged from the data through the constant comparative method [23].

We used of the Atlas-Ti 5.0 software program as a technical aid in the coding stage, enabling to code large amounts of text and share the data among the research team.

### Rigour

The validity and reliability of the conclusions of the analysis were ensured by the following: literal transcription of all interviews, analysis of the data in the full context of the interview during which they had surfaced, constant comparative method and triangulation methods [27,29]. We use triangulation methods to increase the validity and to mitigate biases in the study [30,31]. Thus, data were analyzed by three researchers from different disciplines who examined the phenomena from multiple lenses and possible theories. In addition, we performed data source triangulation conducting in-depth interviews with a theoretical sampling of residents and proxies of different ages, sex and sociodemographic characteristics [30,31].

## Results

The emergent theoretical categories that explained participants’ perceptions are described in Table 4, constituting the basics for developing a substantive theory.

**Table 4:** Codes, subcategories and categories describing perceptions of quality of care

**Codes**	**Subcategories**	**Categories**	**Description**
Good will	Emotional competences	Aspects related to the persons providing care	Professional competences in nursing homes
Affection			
Kindness			
Cheerfulness			
Humor			
Warm care			
Good manners and respect			
Individualized care	Technical competences		
Listening			
Support			
Calm and patience			
No anonymity			
Tact			
Empathy			
Suitable information			
Specific training in Geriatrics			
Round-the-clock care	Health care	Institutional aspects of the care’s process	Aspects related to the institutional quality standards
Specialized centers			
Job stability	Work routines		
No strict rules			
Family participation in care			
Cleaning	Facilities and services		
Recreational activities			

Two main categories emerged from the analysis of the residents’ and family members’ perceptions the quality of nursing-home care, such as aspects related to the persons providing care, or as institutional aspects of the care’s process. But in all cases participants prioritized in their discourses aspects related to the persons providing care rather than institutional aspects of the care’s process. The results are presented beginning with the categories, subcategories and codes. In the interests of achieving a better understanding of the results we have included the most representative verbalizations; the number assigned to each participant follows each quote and the word “proxy” in the case of the discourses of family members.

### Quality defined as aspects related to the persons providing care

All participants included aspects related to the persons providing care as the pillar of quality of care. This category encompassed participants’ conceptualizations of competences related to care in nursing homes. This comprised emotional and technical competences. But throughout their discourses the participants emphasized the importance of emotional competences versus technical competences.

#### Emotional competences

Participants agreed when it came to including good will, affection and kindness on the part of professionals as an essential ingredient of aspects related to the persons providing care:

“Since they have to do things, what I most value is that they go about them with a good will” (P.20).

In the case of residents, there were also differences according to gender. While women felt that, “kind and considerate care provides warmth and affection” (P.11), men talked of kindness, “quality means that people say things in a friendly way” (P. 26).

The residents also included cheerfulness and a sense of humor as an essential attitude for achieving good professional care:

“Laughter is the best therapy” (P.7). “Being cheerful goes a long way because, even though you may not be able to do anything in certain cases, at least you can be a little friendly at such times” (P.25).

In addition, the relatives of residents with cognitive impairment included family-like and warm care:

“Quality is closeness… they should treat residents with family-like familiarity” (P.2 Proxy). “You ought to care for them as if they were your mother or a person close to you” (P.3 Proxy).

All participants agreed in asserting that good manners and respect for the client should be uppermost when caring for residents:

“To my mind, the worst thing of all is bad manners. One of the things that old folks most appreciate is the way in which they are treated” (P.1 Proxy). “I see this as being like a small hotel where people are treated with respect and courtesy, and I feel more at ease” (P.2 Proxy).

#### Technical competences

In the informants’ opinion, professionals had to provide an individualized care:

“They should take the preferences of residents and family members into account” (P.2 Proxy). “Care should be personalized” (P.6 Proxy).

Participants conceptualized as another pillar of aspects related to the personal providing care the professionals’ role of listening and providing support, the result of a close relationship with the residents:

“It’s not that they’re going to get rid of the pain or anything, but rather that they give you encouragement” (P.25).

On the other hand, residents wanted to be attended at a pace that allows them to preserve their functionality:

“Care should be given with calm and lots of patience” (P.23).

Furthermore, the data showed that residents do not like anonymity. All their lives, they have been called by their own names, something that gives them a personal identity in their professional or social environments, which they want to retain: “I like to be called by my name” (P.27). Residents wish that professionals and family members would put a stop to paternalistic attitudes, and cease to regard residents as mere objects and treat them as though they were children:

“They shouldn’t think we’re stupid and treat us like children, because I’m already an adult” (P.23).

To ensure correct interaction, and avoid misunderstandings and conflictive situations between residents with cognitive impairment and professionals, their family members insist that they be attended “with tact” and empathy providing individualized care based on knowledge of the personal needs of each resident:

“It is extremely important that they know their weak points, and guide them to neutral ground” (P.3 Proxy).

In their description of quality, family members attributed higher priority to a close professional-resident relationship than to knowledge:

“With just a word, a smile or a look. That’s more important than the staff knowing a lot” (P.17 Proxy).

Institutionalized elderly are grateful when people enquire and are attentive to them: “Being well treated is when somebody comes over to you and says: Do you need anything? Would you like this or that?… That’s what it’s about, coming and asking you” (P.18). In order for the resident-professional relationship to be satisfactory, it is also important that adequate information be received from the professionals, i.e., “that they tell you what they’re going to do to you” (P.14). This information calms residents and makes them understand that sometimes they have to wait to be attended, and that this may even have therapeutic effects:

“If you call them, then they should tell you…well now we can, now we can’t…they should keep you informed” (P.25).

Female relatives, particularly in the case of residents with severe cognitive impairment, considered that medical and nursing staff had the necessary skills to handle residents by virtue of their training, whereas nursing assistants ought to have better training in geriatrics, which would directly impact on improving the care of and interaction with residents, and on the individualization of care:

“I’d ask for a little more in the way of qualifications” (P.6 Proxy). “I feel that to be able to give quality, professionals must have specific training in older persons” (P.17 Proxy).

### Institutional aspects of the care’s process

The second pillar of the concept of quality which emerged from our analysis of the data was institutional aspects of the care’s process according to organizational technical standards, though this always appeared in a subordinate role, coming after aspects related to the persons providing care.

Unlike family members, residents were less critical and adopt a more conformist view on aspects related to organizational technical quality in nursing homes:

“I’m 93 years old, what am I going to ask here? Everything seems fine” (P. 12).

Among the services that a nursing home must have, family members and three male residents stressed the importance of experienced and fast provision of health care. Family members felt that such a service afforded a sense of security:

“If there’s some kind of problem or something happens at night, there’s always a physician or qualified nurse on call, and that puts my mind at rest” (P.4 Proxy). “One of the important things is 24-hour care” (P.3 Male).

Moreover, family members insist on job stability among professional staff, since constant changes in personnel hinder the relationship of trust between resident and worker. This idea is in line with view that residents have of staff as being a source of support in difficult situations:

“I feel that the same staff should always be there because they know the residents. They (the residents) tell them all about themselves and come to have a lot of trust in them” (P.1 Proxy). “They’re used to old people and know what has to be done in order to deal with them” (P. 17 Proxy).

Within the context of institutional aspects of the care’s process, there were differences according to sex and age. Hence, only male residents included facilities and cleanliness in their perception of quality:

“I’d say that a nursing home has quality on the basis of its staff, building, rooms, services and 24-hour medical care” (P.23). “Facilities are also important, but the personal touch, warmth and tenderness, is more important” (P.7). “The fact that you are well taken care of, that there’s cleanliness, to me that’s quality” (P.26).

With respect to nursing home’s services, the aspect most highly prized by participants was that care was provided on the basis of residents’ needs: “quality is not a matter of great luxury, but rather having one’s needs covered” (P.24). Residents laid emphasis on food needs, putting quality before quantity: “Quantity’s not important, what’s important is the quality of the meals and the way they are presented” (P.14). Furthermore, family members valued the fact that, when it came to attending to the residents, the professional caregivers showed personalized attention to small details:

“They are attentive with my mother, thus when they put her in the chair, they placed a blanket over her knees so that she wouldn’t feel the cold” (P.28 Proxy).

Residents under the age of 75 years valued the fact that there were no strict rules, and that they preserved part of their independence despite their institutionalization:

“The fact of the matter is that I’m neither old nor young, and I feel pretty good, with a strong will to live and fit and active, I can fend for myself, and freedom’s what I like best” (P.9). “We really value the fact that the nursing home’s open and we’re free to go out” (P.5). “If I couldn’t leave the nursing home, I’d feel suffocated; there’d be nothing left for me” (P.24).

Within this view of institutional quality standards, we found that relatives of residents with advanced cognitive impairment demanded the introduction of centers specializing in these types of disorders:

“My husband ought not to be here because, with the illness he has, he shouldn’t be in a nursing home: he should be in a chronic disease unit” (P.6 Proxy).

Something prized as a quality criterion by relatives of residents with cognitive impairment is the fact that they allowed to become involved in decisions affecting their relative’s care:

“As soon as they see me, they say, look, your mother has done this, needs that… My mother wasn’t eating properly and the doctor asked me if I wanted them to fit her with a feeding tube, but I told the doctor, better not, and that, even though she ate very little, I’d prefer it to be without a tube” (P.28 Proxy).

Family members’ idea of institutional aspects of the care’s process was completed by one last component, namely, the presence of recreational activities at the nursing home:

“They should have some time for leisure and recreation” (P.15 Proxy).

## Discussion

Our study is among the few to have used in-depth interviews to ascertain the protagonists’ points of view on how quality of care in nursing homes should be conceptualized, and is the only one conducted at a nursing home in a Mediterranean cultural setting. Furthermore, our study also furnishes a simultaneous analysis of the conceptualizations of older institutionalized persons with and without cognitive impairment, with the perceptions of the former being incorporated through the agency of proxy informants.

From our study emerge the following theories that explain the patterns of relationship among constructs that arise from the discourse of the participants: 1) Aspects related to the persons providing care are the pillar of quality care; 2) professional competences for geriatric care are components of quality, but emotional competences are considered more important than aspects of the care’s process; 3) the participants’ perceptions of quality of care are highly influenced by the traditional ideas about how to provide the best care for older people; and 4) conceptualizations of nursing home are different for residents than for resident’s family members.

The importance of interpersonal relationships as a component of quality of care is a characteristic that tends to emerge in qualitative studies [14,16,32]. Our results are consistent with other qualitative studies, showing that, for nursing-home residents, quality of care consisted of the following professional competences: care tailored to individual needs [16,33]; person-centered care [14,34]; interest in the resident [14]; physical contact; ability to listen [14]; avoidance of tendency to regard residents as mere objects; closeness [16,32]; receive information about care [12-14]; empathy and sympathy [16] and respect for their values, preferences [13,14,34]. There are many voices that stress respect for residents’ choice [9,13,32], however their decision-making capacity is often overlooked by long-term care managers [35].

In contrast with survey-based studies [7,33], our study participants attribute less importance to technical aspects of care than to quality of human relationships. The points of view of professionals also emphasize on tangible aspects (physical needs) rather than in familiar and low tech and treatment [12,36].

In line with previous studies [37], for our participants interpersonal and technical quality criteria of the process of care prevailed over both structural aspects -facilities, cleanliness, independence- and outcome-related aspects -survival, falls, etc.- [1,10].

Notwithstanding this, at least in Spain, most of the institutions mainly include in their quality assessment items related to technical issues, while usually the interpersonal aspects are optional items [1,38]. Maybe, and although that is changing now, this could be due to the fact that accreditation and evaluation of long term care public institutions in Spain has traditionally been in a similar way that health services where technical equipment plays a pivotal role, and the voice of clients are not listened to.

As in other studies [14], for our residents professional caregivers’ training was not an important matter; however, for relatives of residents with cognitive impairment staff training indicators were essential elements for overall health care quality, highlighting that better qualifications can lead to improved skills. Family members considered themselves as experts in the care of their loved ones, and from this position were able to question the basic care dispensed by junior nursing staff, though not the diagnoses made or care given by mid-level or senior staff. This is a new aspect that has not been highlighted in previous studies.

Although the number of nursing professionals has been associated with quality of care [1,2], our informants make no reference to these aspects. In contrast, they consider that job security of the staff and the professional expertise were essential to person-centered care and to quality of care, since high staff turnover was considered a barrier for a relationship of trust and confidence between residents and professionals.

We consider that cultural stereotypes can influence on care conceptualizations. In contrast with other studies conducted in countries such as USA, where care is more consumer-oriented, and in which formal care systems for the elderly people are commonly viewed in a positive light [24,39], family members considerer the family as the best providers of care for older people and the institutionalization as the last option of care. According with this conceptualization of the nursing home as a substitute of family, proxy informants demanded more participation in the decision-making about the care of their relatives.

As a result, their perceptions about aspects related to the persons providing care includes a family-like relationship, based on closeness, person-centered care and respect for the resident’s autonomy. Similarly, in other studies, residents [32,33] and professionals [36] state that the ideal care setting is a “homelike” atmosphere.

In the opinion of family members, quality of care includes the presence of 24-hour health care as another basic pillar of quality, but they focused health care exclusively on physicians and nurses, and neglected the other components of the team. Other studies have also underscored the importance that has early care has for clients when they have a problem [16]. However, this aspect of the assistance did not emerge in the discourse of all residents. These differences in conceptualization between proxies and residents may possibly be due to the fact that, in our study, the proxies were relatives of persons who were suffering from cognitive impairment, namely, persons requiring more health care.

Perceptions of relatives tend to resemble those clients of private health services, being more critical and demanding than residents in their assessments of quality. Conversely, and according with other studies [40,41] the resident’s perceptions of publicly-funded nursing homes reflect conformism and passive acceptance of prevailing standards. These differences might be due to the fact that family members are usually younger than residents, and they are accustomed to evaluate services and have no fear of reprisals, as described in other studies [14]. In our opinion, this is sufficient reason for considering the opinion of proxies when assessing the quality of care in nursing homes.

### Limitations

The size of our theoretical sample, albeit insufficient to ensure external validity in terms of other empirical research models [29], was nevertheless sufficient to provide great analytical richness, by including participants of widely different sociodemographic backgrounds.

Although the use of proxies for presenting the views of people with cognitive impairment may have some limitations, it has been considered as the best alternative to analyze care when patients cannot do by themselves [18,42].

Furthermore, it is important to bear in mind that data were collected at a publicly-owned nursing home, and results should be generalized to other types of nursing homes with caution, particularly to those facilities where the end-user has to pay for the entire range of services on offer, and thus feels entitled to other rights as a client.

## Conclusion

Our results are useful for practitioners and managers of nursing homes, since they analyze quality of care in nursing homes from the point of view of those for whom it is intended, and highlight that, for residents of nursing homes, less visible aspects of quality of care as emotional needs attention may be more relevant than more tangible aspects traditionally used as quality standards.

Multiple viewpoints (clinicians, managers, policymakers and residents) should be taken into account in the assessments of the quality of care in nursing homes. In this way, on the basic of patient centered approach of quality, the understanding of the residents and their proxies’ viewpoints might help to individualize the care, and might be useful to analyze this quality of care from a holistic perspective.

## Abbreviations

USA: United States of America.

## Competing interests

The authors of this study declare that they have no competing interests.

## Authors’ contributions

All authors have contributed substantially to the manuscript. In particular, BRM and VMV that were the principal investigators contributed to conception, design, analysis and interpretation of data. BRM, MMA, BCM and BNP contributed to drafting of the manuscript and revising it critically. Also, VMV coordinated the study and reviewed the work done in the study. All authors provided final approval of the manuscript submitted.

## Authors’ information

1. BRM Degree: PhD. MSc. Professor Assistant of Gerontology.

2. MMA Degree: MSc. Professor Assistant of Sociology.

3. BCM Degree: MSc.

4. BNP Degree: PhD. MSc. Professor Assistant of Nursing.

5. VMV Degree: MD. Professor of Nursing.

## Pre-publication history

The pre-publication history for this paper can be accessed here:

http://www.biomedcentral.com/1471-2318/13/65/prepub
